# Driving Fatigue Detection from EEG Using a Modified PCANet Method

**DOI:** 10.1155/2019/4721863

**Published:** 2019-07-14

**Authors:** Yuliang Ma, Bin Chen, Rihui Li, Chushan Wang, Jun Wang, Qingshan She, Zhizeng Luo, Yingchun Zhang

**Affiliations:** ^1^Intelligent Control & Robotics Institute, College of Automation, Hangzhou Dianzi University, Hangzhou, China; ^2^Department of Biomedical Engineering, University of Houston, Houston, Texas, USA; ^3^Guangdong Provincial Work Injury Rehabilitation Hospital, Guangzhou, China

## Abstract

The rapid development of the automotive industry has brought great convenience to our life, which also leads to a dramatic increase in the amount of traffic accidents. A large proportion of traffic accidents were caused by driving fatigue. EEG is considered as a direct, effective, and promising modality to detect driving fatigue. In this study, we presented a novel feature extraction strategy based on a deep learning model to achieve high classification accuracy and efficiency in using EEG for driving fatigue detection. EEG signals were recorded from six healthy volunteers in a simulated driving experiment. The feature extraction strategy was developed by integrating the principal component analysis (PCA) and a deep learning model called PCA network (PCANet). In particular, the principal component analysis (PCA) was used to preprocess EEG data to reduce its dimension in order to overcome the limitation of dimension explosion caused by PCANet, making this approach feasible for EEG-based driving fatigue detection. Results demonstrated high and robust performance of the proposed modified PCANet method with classification accuracy up to 95%, which outperformed the conventional feature extraction strategies in the field. We also identified that the parietal and occipital lobes of the brain were strongly associated with driving fatigue. This is the first study, to the best of our knowledge, to practically apply the modified PCANet technique for EEG-based driving fatigue detection.

## 1. Introduction

As a leading factor in traffic accidents, driving fatigue accounts for 14%–20% of motor vehicle accidents that cause serious injuries and fatalities to human life [1]. Consequently, growing attention has been paid to driving safety in recent years. Driving safety is thought to be affected by multiple factors, including monotonous environments, sleep deprivation, chronic sleepiness, drug and alcohol use [2, 3], wherein the most common factor is driving fatigue [4, 5]. In such situation, drivers are most likely to fall asleep and drive unconsciously, which is not only a serious threat to the driver's own life and safety, but also a serious threat to the involved pedestrians and other vehicles. Therefore, detection of driving fatigue is of great importance to reduce the frequency and severity of traffic accidents [6].

In recent years, a variety of methods have been proposed to detect driving fatigue. For instance, Hiesh and Tai developed an infrared light-based digital signal processing (DSP) embedded system to capture driver's face and detect driving fatigue by identifying the opening and closing of eyes [7]. In another study, a calculation method named as the improved percentage of eyelid closure over the pupil overtime (PERCLOS) was employed as a standard criteria to judge whether the driver was tired or not [8]. Although this method is convenient in fatigue detection, it is vulnerable to environmental illumination such as the brightness, resulting in poor detection performance [9]. Later on, a sensor-based device called the steering wheel monitoring system (SAM) was developed to detect driving fatigue by monitoring the abnormal movement of the vehicle's steering wheel [10]. Although detection of steering wheel features good real-time performance and low cost, it also suffers poor anti-interference ability and low reliability [11].

Recently, fatigue detection based on physiological signals such as electroencephalogram (EEG), electrocardiogram (EOG), electromyogram (EMG), and electrocardiogram (ECG) signals has been increasingly investigated in this field [4, 12–19]. Among them, EEG has some major assets in detecting driving fatigue due to its high temporal resolution, high portability, and good sensitivity to fatigue. With this in mind, a variety of studies have attempted to perform EEG-based classification using different signal processing techniques to accurately detect the fatigue during driving. For instance, Yang et al. implemented the detection of driving fatigue using information fusion and dynamic Bayesian neural network [20]. In another study, Zhao et al. demonstrated that wavelet packet can be used to extract features from EEG signal and classify the driving condition by a support vector machine (SVM) [21]. However, the EEG-based fatigue detection during driving is still faced with challenges. For instance, EEG signals are usually collected with low signal to noise ratio (SNR), which requires large effort in preprocessing raw EEG data. Moreover, the widely employed EEG-based classification techniques depend heavily on handcrafted features, which is time-consuming and highly relies on skilled person in the domain before predictions are performed. Therefore, there is a clear need to develop a new strategy to improve the robustness and efficiency of EEG-based driving fatigue detection.

As a simplified deep learning model based on convolution neural network (CNN), principal component analysis network (PCANet) has been developed and widely used for feature extraction in two-dimensional image processing [22]. Referring to a previous study that performed EEG-based lie detection [23], PCANet was proved to be highly effective in classification problem as it automatically extracted features from multichannel EEG data based on the deep learning technique rather than extracting handcrafted features in conventional ways. However, PCANet may be subject to the phenomenon of dimension explosion when the dimensionality of input data is large, which dramatically increases the complexity and cost of computation, rendering it more difficult to be effectively employed in multichannel EEG signal processing.

To address this challenge, this study aimed to adapt the PCANet algorithm to enhance the efficiency of conventional EEG-based driving fatigue detection by incorporating the principal component analysis (PCA) with the PCANet technique. Specifically, PCA is used to preprocess the multichannel EEG signals and reduce the dimensionality of data prior to the PCANet processing. The performance of the proposed modified PCANet method in driving fatigue detection was evaluated by comparing to traditional PCANet and other conventional features extraction methods widely used in driving fatigue detection [4].

## 2. Methods and Material

### 2.1. General Structure and Purpose

**Figure 1:**
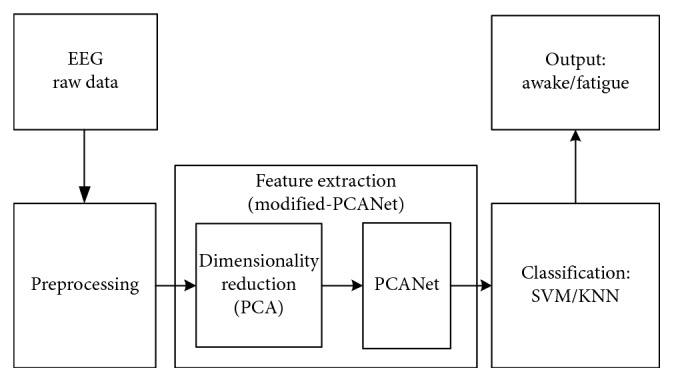
The overall schematic for the proposed EEG-based driving fatigue classification.

The main structure of this study is demonstrated in Figure 1. EEG data was first collected in a simulated driving environment, and followed by conventional signal preprocessing procedures. PCA was then used to reduce the dimensionality of the preprocessed signals, which were fed into the PCANet for feature extraction. Finally, the extracted features were classified using a support vector machine (SVM) and a *K*-Nearest Neighbors (KNN) classifier with respect to the driving condition.

### 2.2. Participants

Six male volunteers (right handed, aged 25.00 ± 2.00 years) with valid driver's licenses were recruited to participate in the experiment. All subjects were physically and psychologically healthy without any sleep disorders. The experiment was approved by the research ethics board of Guangdong Provincial Work Injury Rehabilitation Center and performed in accordance with the Declaration of Helsinki. Each subject was fully informed about the purpose of the research and provided written, informed consent prior to the start of the experiment.

### 2.3. Experiment Design and Data Acquisition

**Figure 2:**
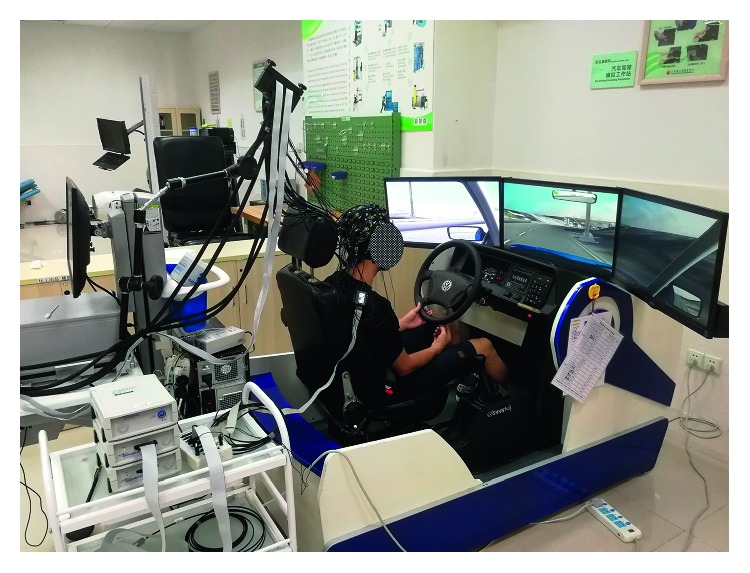
The setup of the experiment, including the driving platform and EEG recording system.

A 32-channel EEG acquisition system (Brain Products GmbH, Germany) was utilized to collect EEG signals with the sampling frequency set to 500 Hz. EEG electrodes were placed on the scalp according to the international 10–20 standard system. An advanced driving simulation system (Shanghai Infrared Automobile Simulator Driving Equipment Co., Ltd., China) was used in this study to simulate a real driving environment. Briefly, the driving simulation system could imitate the real driving scenarios with dynamically changing representations of the car and surrounding traffic. As shown in Figure 2, the driving simulation system consisted of a fixed car steering wheel, the brake and accelerator pedals, three large screens, a high-performance computer, driving simulation software, and a multifunctional data acquisition board. This system can be adapted to measure the EEG signal in different driving states in real time.

All participants were given sufficient time to practice and get familiar with the driving simulation system prior to the experiment. Datasets in two states were collected for each subject in this study, including the awake state and fatigue state. To collect the data in the awake state, all subjects were required to maintain a natural and adequate sleep for about 8 hours during the night before the experiment. EEG data was collected at 9 a.m. on the next day for about 30–60 minutes while subjects were driving in the simulated environment. To collect the data for the fatigue state, all subjects were allowed to sleep for only 4 hours during the night before the experiment. The EEG data was then measured at 9 a.m. for 30 to 60 minutes while subjects were driving in the simulated environment. Specifically, the driving path was set to relatively long, straight with smooth curves and almost no pedestrian to increase the drowsiness of the subjects in fatigue group, while the path was set to relatively complicated to avoid the drowsiness of the subjects in the awake group. During the recording, an observer seated 2 meters beside the subjects monitored the subject's behavior without causing any disturbance to the subjects. The observer decided whether the subject was in a fatigue state or an awake state by observing the subject's drowsy signs (more than two seconds eye closure and head nodding, large deviation off the road) [4, 24]. Data recording was terminated 30 minutes after the subjects began to show fatigue signs. If the subject did not show any sign of drowsiness after 60 minutes of measurement, the experiment was terminated and data from the subject were excluded from further analysis. The experiment was conducted in a quiet, undisturbed laboratory with ambient temperatures around 22°C.

### 2.4. Data Preprocessing and Analysis

All the data analyses in this study were implemented using MATLAB (2014a, MathWorks, Natick, Massachusetts). 20-minute EEG signals in each state were selected for analysis. The raw multichannel EEG signals were first downsampled from 500 Hz to 200 Hz. A third-order bandpass filter (0.1–45 Hz) was then applied to remove artifacts such as slow drift, high-frequency noise, and powerline interference. The 20-minute preprocessed EEG data for each state were then segmented by a 10-second time window, resulting in 120 samples for each state and each subject. It is worthy of noting that in this study one sample was a two-dimension matrix (32 channels × 2000 data points). Overall, a total of 1440 samples were obtained from all subjects for classification (720 samples for awake and 720 samples for fatigue).

### 2.5. PCA Dimensionality Reduction and Extraction of Main Features

In this study, the proposed modified PCANet consisted of two steps, PCA-based dimensionality reduction and PCANet-based feature extraction (Figure 1). To overcome the dimension explosion problem caused by conventional PCANet, PCA was first employed to reduce the dimensionality of each EEG sample. Briefly, for a given EEG sample data (32-channel × 2000-point), the PCA transformed the data to 2000 linearly uncorrelated components known as principal components, ordered by the amount of variance of the original data that each component accounts for [25, 26]. By keeping the first *r* components with the largest variances and removing the remaining components, the size of original sample data could be reduced to 32 by *r*. In order to keep the original signal information as intact as possible, we kept the previous *r* components that accounted for at least 99% of the original signal as threshold, which was 20 for all samples in this study. Therefore, size of each sample was reduced to 32 by 20 after PCA optimization, and the optimized EEG data was analyzed using PCANet for feature extraction in the next step.

### 2.6. PCANet-Based Feature Extraction

As we introduced previously, PCANet is widely used in 2-D image processing, such as face recognition [27]. In this study, each optimized EEG sample was treated as a 2-D data matrix (32 × 20) and fed into PCANet for feature extraction.

**Figure 3:**
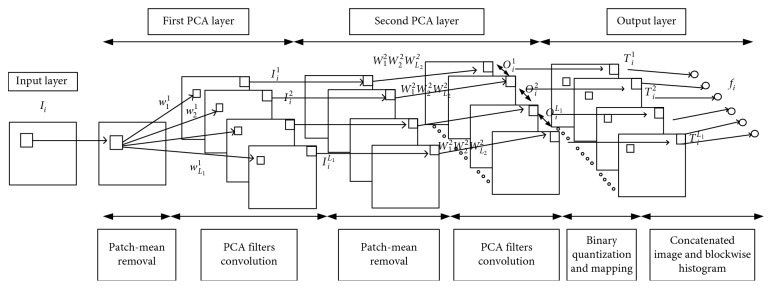
The PCANet network structure.

The workflow of the PCANet network is shown in Figure 3, and details of the algorithm can be found in [22, 28]. In brief, the PCANet consists of two PCA-based filtering layers and an output layer that includes processing of binary hashing and blockwise histogram.

Assume we have *N* input samples after the EEG data preprocessing. Here, each EEG sample is treated as a two-dimensional signal of size *m* × *n* (channel number × sample number). Given an input EEG sample *X*_*i*_, a sliding window of size *k*_1_ × *k*_2 _ is used to centralize the EEG sample by subtracting the mean value of each window's data from the corresponding window. Each centralized window is further vectorized into a single column, from which the input EEG sample is converted to a new 2-D matrix ${\bar{X}}_{i}\;$ consisting of all centralized windows. The same processing is applied to all input EEG samples ({*I*_*i*_}_*i*=1_^*N*^) to obtain the following data structure:

(1) $$\begin{array}{c} X=\left[{\bar{X}}_{1},{\bar{X}}_{2},\ldots ,{\bar{X}}_{N}\right]\varepsilon R^{k_{1}k_{2}\times Nc}, \end{array}$$

where *c*=(*m* −  *k*_1_+1)( *n* −  *k*_2_+1) is the number of columns in ${\bar{X}}_{i}$.

The new vectorized matrix *X* is then used to perform the PCA filtration in the first layer. Specifically, the covariance matrix of *X*, denoted as *X*_cov_=*XX*^T^/*Nc*, is computed and applied to select the eigenvectors corresponding to *L*_1_ principal eigenvalues as PCA filters *W*_*l*_^1^. For the *i*th EEG sample, the output of the first PCA layer is then given by the convolution of the input EEG sample and the PCA filters:

(2) $$\begin{array}{c} I_{i}^{l}={\bar{I}}_{i}\;*\;W_{l}^{1},\;i=1,2,\ldots ,N\text{ and }l=1,2,\ldots ,L_{1}, \end{array}$$

where *I*_*i*_^*l*^ denotes the *l*th output of the *i*th EEG sample and ${\bar{I}}_{i}$ is the zero‐padded form of *I*_*i*_ to ensure the same size of *I*_*i*_^*l*^ and *I*_*i*_.

The second PCA layer is similar to the first layer. The output of the first PCA layer is centralized with the same sliding window and applied to select the eigenvectors corresponding to *L*_2_ principal eigenvalues as PCA filters *W*_*p*_^2^. The output of the second PCA layer is given as

(3) $$\begin{array}{c} O_{i}^{l}\;\dot{=}\;{\bar{I}}_{i}^{l}\;*\;W_{p}^{2},\;i=1,2,\ldots ,N\text{ and }p=1,2,\ldots L_{2}. \end{array}$$

With the result obtained from the filtrations of two PCA layers, the output of the PCANet (*T*_*i*_^*l*^) is further processed by binary hashing as

(4) $$\begin{array}{c} T_{i}^{l}=\overset{L_{2}}{\underset{p=1}{\sum }}2^{p-1}H\left({\bar{I}}_{i}^{l}\;*\;W_{p}^{2}\right),\;\;l=1,2,\ldots ,L_{1}, \end{array}$$

where *H*(·) is a Heaviside step function that sets positive values as one and zero for others.

Finally, for the *i*th input EEG sample, each of the *L*_1_ components in *T*_*i*_^*l*^ is partitioned into *B* blocks. The histogram (with 2^*L*_2_^ bins) of the decimal values in each block is computed and concatenated into one vector represented as Bhist (*T*_*i*_^*l*^). The PCANet-derived feature of the *i*th EEG sample is then denoted as

(5) $$\begin{array}{c} f_{i}={\left[\text{Bhist}\left(T_{i}^{1}\right),\ldots ,\text{Bhist}\left(T_{i}^{L_{1}}\right)\right]}^{\text{T}}\in R^{\left(2^{L_{2}}\right)L_{1}B}. \end{array}$$

The PCANet processing is applied to each EEG sample for feature extraction.

### 2.7. Classification

Support vector machine (SVM) and *K*-Nearest Neighbors (KNN) were employed as classifiers in the classification of awake and fatigue states for each subject. The performance of each classifier was evaluated using a 10-fold cross-validation strategy. At each iteration, 90% of the samples were randomly selected as the training set, and the accuracy, defined as the ratio between correct predictions and the total number of predictions, was computed on the remaining 10% of the samples, the testing set. Accuracies among the 10 steps of the cross validation were then averaged, yielding the mean accuracy for each subject. To evaluate the superiority of the proposed method, the obtained accuracy was compared to the performance obtained from the traditional PCANet method and two commonly used feature extraction methods, i.e., the power spectral density (PSD) [29, 30] and wavelet packet decomposition (WPD) [31].

In this study, the PSD features of each EEG channel in a segmented EEG sample (32-channel × 2000-point) were estimated through Short-Time Fourier transform (STFT) with a 128-point Hanning window and 50% overlap rate. For each EEG channel, the PSD feature of a specific frequency band was computed by averaging all squared magnitude values of STFT within the corresponding frequency range. In this study, five typical EEG bands were investigated, including delta (0.1–4 Hz), theta (4–8 Hz), alpha (8–13 Hz), beta (13–20 Hz), and gamma (20–45 Hz). This resulted in 160 PSD features for each EEG sample (5-band × 32-channel).

The WPD features of each EEG channel in a segmented EEG sample (32-channel × 2000-point) were calculated based on discrete wavelet decomposition (DWT). In brief, the DWT decomposed the selected EEG signal into a number of layers by filtering the signal with quadrature mirror filters (a low-pass filter and a high-pass filter). The output of each layer was a series of detail coefficients (from the high-pass filter) and approximation coefficients (from the low-pass filter), which were extracted as features for classification [32]. In this study, we decomposed each EEG channel data (2000 points) with a 3-layer “Daubechies” wavelet, resulting in 8 groups of coefficients (256 points). Therefore, there were in total 65536 (8-groups × 256-point × 32-channel) features extracted for each EEG sample.

## 3. Results

**Figure 4:**
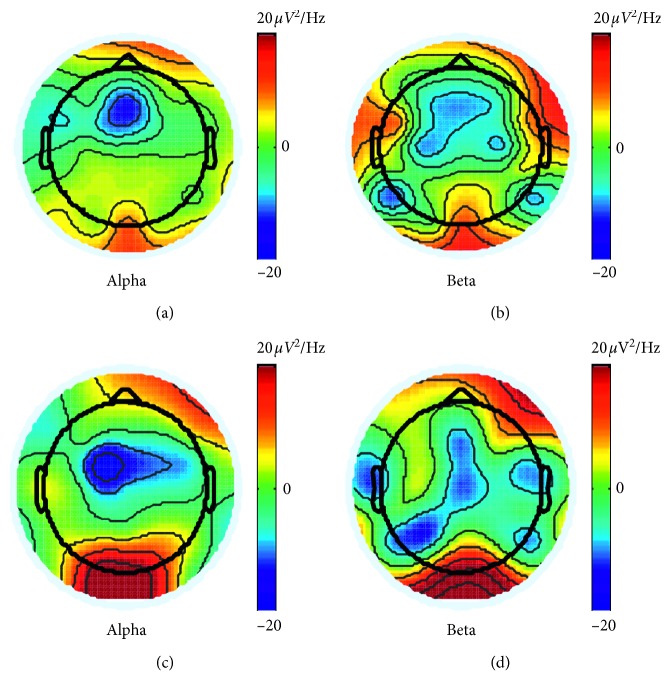
PSD distributions of alpha and beta bands for awake (a, b) and fatigue (c, d) states.

Referring to previous studies [4, 24], it was found that the alteration in brain regions during awake and fatigue states were more prominent at the parietal lobe at alpha and beta frequency bands [33, 34].  Figure 4 shows the group-averaged PSD distribution of the relevant EEG signals in alpha (8–13 Hz) and beta (14–20 Hz) bands in awake and fatigue states. The PSD values were computed based on the average of all 10-second EEG samples in each state for each subject. In brief, in the fatigue state, the PSD in the parietal and occipital lobes of the brain was more pronounced compared to the PSD distribution in the awake state.

**Figure 5:**
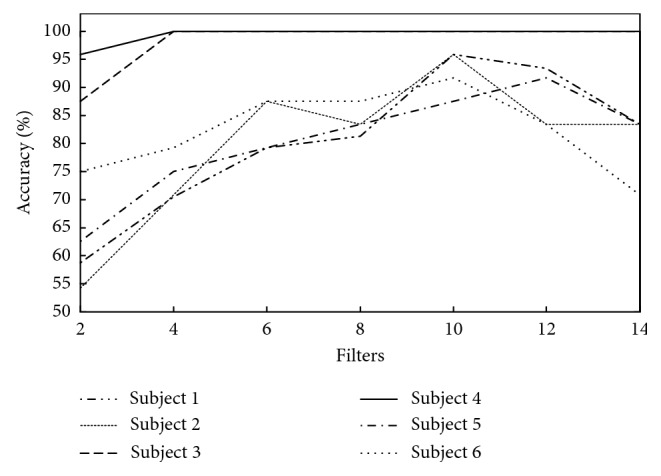
The classification performance of different PCA filter numbers when using SVM.

In order to determine the optimal number of PCA filters (*L*_1_ and *L*_2_) when using the PCANet, the classification performance varied with the number of PCA filters was acquired for each subject.  Figure 5 shows the variation of classification performance for each subject when using a SVM, with number of PCA filters increasing from 2 to 14 for both layers, respectively. Overall, for most subjects (except sub. 3 and sub. 4), the classification performance was enhanced as the filter number increased, and gradually decreased when filter number was over 10 or 12.

**Figure 6:**
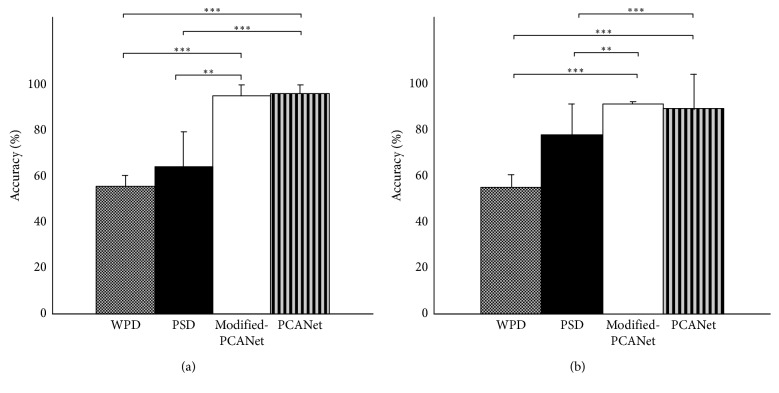
The average accuracies obtained by SVM (a) and KNN (b) when using different features extracted through WPD, PSD, modified PCANet, and PCANet. “*∗∗*” denotes significantly different from controls (*p* < 0.005). “*∗∗∗*” denotes significantly different from controls (*p* < 0.0001).

Additionally, the performance of classification using two classifiers and various feature extraction strategies, including the traditional PCANet, WPD, PSD, and the proposed modified PCANet method, is shown in Figure 6 and summarized in Tables 1 and 2. Overall, for both classifiers, when selecting the optimal PCA filter number for each individual subject, the traditional PCANet and the proposed modified PCANet method achieved better performance in the classification of awake and fatigue states. The result of the paired-*t* test between four feature extraction methods revealed that the PCANet-based methods significantly outperformed the other two methods (*p* < 0.005), as shown in Figure 6 and Table 2. Although no significant difference in classification accuracy was observed between the traditional PCANet and the modified PCANet, the time used in the feature extraction, model training, and testing was drastically reduced when using the modified PCANet method, indicating the high efficiency of this method (Table 3).

In addition, the area under the curve (AUC) of the receiver operating characteristic (ROC) curve [35], which evaluates how well a model separates the groups being classified, was employed to assess the performance of different feature extraction strategies. As summarized in Tables 1 and 2, for both classifiers, the AUC values obtained from the traditional PCANet and the proposed modified PCANet method are significantly higher compared to PSD and WPD features (*p* < 0.005). Similarly, no significant difference in AUC values was observed between the traditional PCANet and the modified PCANet.

## 4. Discussion

This study sought to validate the feasibility of using modified PCANet to enhance the performance of EEG-based driving fatigue detection. The neuronal electrical activity was recorded using EEG in a simulated driving environment with subjects experienced both awake and fatigue states. We employed PCA to alleviate the dimension explosion caused by PCANet before classification. The experimental results indicated a significantly enhanced performance in the fatigue detection compared to the traditional PCANet and other conventional approaches.

Alterations in low and high frequency bands were previously observed by EEG in the drowsy state [36]. In summary, investigations that included the transition from awake to sleepy states have demonstrated an increase in the alpha rhythm [24]. The alteration of the alpha band during drowsiness in both simulated and actual driving conditions was also reported in a previous study [37]. In this study, we compared the PSD between both states for alpha and beta frequency bands and found an increased PSD at occipital and parietal areas in both alpha and beta bands (Figure 4). This finding is in line with the results from these studies, demonstrating the possibility of using EEG as a portable and reliable approach to monitor and detect the driving fatigue.

In order to monitor the brain state during driving, it is of great importance to achieve high accuracy and reliability in detecting the driver's fatigue state. With high classification accuracy between the awake and fatigue states achieved by the modified PCANet approach, our study proved the usefulness of EEG to study driving fatigue. In particular, the substantial increase in classification accuracy using the proposed method, compared to conventional feature extraction methods, offers a new perspective to deal with classification problem when using multichannel biosignals such as EEG and EMG signals. It is noteworthy that PCA was necessarily adopted before PCANet was employed to extract features in this study. As shown in Table 3, the proposed modified PCANet method remarkably reduced the time for the classification while maintaining a comparable performance relative to the traditional PCANet approach. This provides evidence that PCA is able to alleviate the curse of dimensionality induced by PCANet, reducing the computational cost when using the traditional PCANet. By taking this great advantage, the proposed method is considered a more effective strategy in a practical scenario such as monitoring driving fatigue in real time. In addition, the components compressed by PCA retains the main characteristics of original signals, which is the inherent benefit offered by PCA. The pre-refined signals can be further improved by PCANet to achieve the significantly enhanced classification accuracy. Particularly, the classification performance across all subjects not only exhibited high accuracy, but also yielded lower variance, demonstrating the good robustness of the proposed method.

Despite the improvement achieved in this study, there are still several limitations in this study. Firstly, only off-line analysis and small sample size were elected in this study. Real-time fatigue classification should be conducted on larger population base in the future to validate the potential of the proposed approach in actual driving environment. In addition, two-layer PCA structure was applied for feature extraction in this study, and 8–12 filters were considered optimal setting for achieving best performance. Apparently, the number of PCA filters within each layer affected the quality of feature extraction, which significantly affected the performance and efficiency of the fatigue classification. Further investigation on how to automatically select the best filter number for each subject is required. Finally, in the present study we solely focused on adapting a deep learning-based technique to the conventional EEG-based driving fatigue classification and provide a new perspective to deal with classification problem when using multichannel biosignals. Although compelling result was achieved in current study, it is expected that future work will evaluate and employ the state-of-the-art algorithms to enhance the performance of this application. Even though the mentioned limitations may prevent us drawing a solid conclusion, the preliminary results demonstrate the capability of the proposed PCANet-based algorithm to monitor and detect the driving fatigue in advance so that it can prevent motor vehicle collision caused by driver drowsiness.

## 5. Conclusion

In this paper, a novel feature extraction strategy incorporating the PCA and PCANet techniques was proposed to enhance the classification performance in EEG-based driving fatigue detection. Significantly enhanced classification performance was achieved using the proposed modified PCANet method compared to the traditional PCANet algorithm and two conventional feature extraction strategies. Additionally, the power spectrum analysis of EEG signals indicated a higher power alteration at occipital and parietal areas in alpha and beta bands. The findings in this study not only demonstrated the effectiveness of using EEG to monitor driving fatigue but also provided a new perspective to adapt a novel machine learning algorithm to investigate the nature of philological signals.

## Figures and Tables

**Table 1:** The classification performance using different feature extraction approaches.

Methods	Classifiers	Classification performance	
		Accuracy (%)	AUC
WPD	SVM	55.42 ± 5.09	0.51 ± 0.11
	KNN	54.00 ± 5.00	0.46 ± 0.08

PSD	SVM	64.44 ± 15.06	0.55 ± 0.12
	KNN	76.00 ± 13.00	0.55 ± 0.08

Modified-PCANet	SVM	95.14 ± 4.87	0.97 ± 0.04
	KNN	89.00 ± 10.00	0.89 ± 0.12

PCANet	SVM	96.00 ± 4.00	0.98 ± 0.03
	KNN	87.00 ± 15.00	0.91 ± 0.10

**Table 2:** The summary of the statistical analysis (*t*-test) of the classification performance between all feature extraction methods.

Methods	Classifiers	*p* values	
		Accuracy (%)	AUC
Modified-PCANet-WPD	SVM	1.36*e* − 07	1.02*E* − 04
	KNN	6.15*e* − 05	5.18*E* − 04

Modified-PCANet-PSD	SVM	0.0023	2.24*E* − 04
	KNN	0.0018	3.10*E* − 03

Modified-PCANet-PCANet	SVM	0.3541	0.6612
	KNN	0.6823	0.2884

**Table 3:** The average time (seconds) used in the feature extraction, model training, and testing between the traditional PCANet and the proposed modified PCANet method.

Steps	Methods		Number of PCA filters					
			2	4	6	8	10	12
Features extraction	Modified-PCANet		0.89	1.46	2.18	2.91	4.22	7.71
	PCANet		125.43	219.94	320.39	449.50	651.95	1202.40

Model training	Modified-PCANet	SVM	0.05	0.70	5.67	10.34	13.25	15.65
		KNN	0.02	0.30	2.20	4.10	5.12	6.15
	PCANet	SVM	7.02	126.66	302.52	736.79	1813.40	3606.80
		KNN	2.70	49.01	118.60	285.04	697.46	1387.23

Model testing	Modified-PCANet	SVM	0.25	0.26	0.26	0.28	0.34	0.39
		KNN	0.10	0.12	0.13	0.15	0.13	0.16
	PCANet	SVM	1.16	2.94	5.34	11.10	20.84	35.75
		KNN	0.45	1.14	2.10	4.22	8.02	14.06

## Data Availability

The data used to support the findings of this study are available from the corresponding author upon request.
